# Pharmacological Effects of Antioxidant Mycosporine-Glycine in Alleviating Ultraviolet B-Induced Skin Photodamage: Insights from Metabolomic and Transcriptomic Analyses

**DOI:** 10.3390/antiox14010030

**Published:** 2024-12-29

**Authors:** Kai Wang, Ling Qin, Huan Lin, Mengke Yao, Junhan Cao, Qing Zhang, Changfeng Qu, Yingying He, Jinlai Miao, Ming Liu

**Affiliations:** 1 Key Laboratory of Marine Drugs, Ministry of Education, School of Medicine and Pharmacy, Ocean University of China, Qingdao 266003, China; wk2303@stu.ouc.edu.cn; 2 Qingdao Key Laboratory of Marine Natural Products Research and Development Laboratory, First Institute of Oceanography, Ministry of Natural Resources, Qingdao 266061, China; qinling@fio.org.cn (L.Q.); linhuan@fio.org.cn (H.L.); ymk@stu.ouc.edu.cn (M.Y.); caojunhan@stu.ouc.edu.cn (J.C.); zhangqing@fio.org.cn (Q.Z.); cfqu@fio.org.cn (C.Q.); heyinging@fio.org.cn (Y.H.); 3 Laboratory for Marine Drugs and Bioproducts, Qingdao Marine Science and Technology Center, Qingdao 266237, China

**Keywords:** mycosporine-glycine, antioxidant properties, UVB exposure, skin photodamage, oxidative stress, collagen protection, skin metabolomics, skin transcriptomics

## Abstract

Mycosporine-glycine (M-Gly), a member of the mycosporine-like amino acid (MAA) family, is known for its potent antioxidant and anti-inflammatory properties. However, its in vivo efficacy in alleviating acute skin photodamage, primarily caused by oxidative stress, has not been well explored. In this investigation, 30 female ICR mice were divided into four groups: a control group and three Ultraviolet B (UVB)-exposed groups treated with saline or M-Gly via intraperitoneal injection for 30 days. At the end of the experiment, UVB exposure caused erythema, wrinkling, collagen degradation, and mast cell infiltration in mouse dorsal skin. M-Gly treatment improved skin appearance and reduced mast cell numbers, while also elevating antioxidant levels, including superoxide dismutase (SOD), catalase (CAT), and glutathione (GSH). Furthermore, M-Gly reduced inflammatory cytokines, such as tumor necrosis factor-alpha (TNF-α), interleukin-6 (IL-6), and IL-1β, typically upregulated after UVB exposure. M-Gly also protected skin collagen by upregulating type I procollagen and decreasing MMP-1 levels. Skin metabolomic profiling identified 34 differentially abundant metabolites, and transcriptomic analysis revealed 752 differentially expressed genes. The combined metabolomic and transcriptomic data indicate that M-Gly’s protective effects may involve the regulation of ion transport, cellular repair, metabolic stability, collagen preservation, and the Nrf2/HO-1 pathway. These findings highlight M-Gly’s potential as an endogenous antioxidant for protecting skin from UVB-induced damage.

## 1. Introduction

Ultraviolet (UV) radiation is generally classified into three types based on wavelength: long-wave UVA (320–400 nm), medium-wave UVB (280–320 nm), and short-wave UVC (200–280 nm) [1]. Owing to extended contact with environmental elements, the skin emerges as the organ most susceptible to damage from UV rays. Among these, UVA and UVB are the primary damaging components of solar UV radiation. Exposure to them can lead to immediate effects such as skin reddening, swelling, and sunburn, as well as trigger inflammation and suppress the immune response. Moreover, they play a role in long-term skin damage, speeding up the aging process and resulting in the appearance of wrinkles and sagging skin [2]. Compared to UVA, the shorter wavelength UVB radiation possesses higher energy [3], which induces the absorption of UV light by non-DNA chromophores within cells. Consequently, this leads to the overproduction of reactive oxygen species (ROS), exerting significant cytotoxic effects and genotoxic impacts on skin cellular health [4]. Nuclear factor erythroid 2-related factor 2 (Nrf2) is an essential transcription factor that regulates the endogenous antioxidant system to mitigate the advancement of numerous diseases associated with oxidative stress [5,6,7]. The Nrf2-dependent pathway forms the primary cellular defense mechanism against oxidative damage [8]. Heme oxygenase-1 (HO-1), a key cytoprotective enzyme induced via the Nrf2 pathway, is pivotal in preserving cellular homeostasis through the degradation of heme [9,10]. In addition to HO-1, other antioxidant enzymes are also pivotal in maintaining cellular redox homeostasis to counter oxidative damage, including NAD(P)H: quinone oxidoreductase 1 (NQO1), catalase (CAT), and superoxide dismutase (SOD) [11]. UV radiation can generate an overabundance of ROS, which may exhaust antioxidant reserves. This depletion can disrupt the functionality of the antioxidant defense system, resulting in a disruption of the body’s redox balance [12]. This imbalance leads to metabolic disturbances within diverse skin cell types, which become evident through the development of lax skin, wrinkles, dryness, increased pigmentation, skin thickening, and the breakdown of collagen [13].

In recent years, the focus on acute skin photodamage has intensified, largely due to the synergistic impact of environmental stressors, notably the depletion of the ozone layer [14]. Severe skin photodamage can lead to conditions like actinic keratosis, photoaging, and skin cancer [15]. As a result, effectively preventing and treating acute photodamage has become a major focus of public concern and a significant challenge in clinical practice [16]. Compared to physical or chemical drug interventions, antioxidants are widely recognized as a safer and more universally applicable approach for the prevention and treatment of skin photodamage. However, the antioxidants currently used to treat skin photodamage are primarily limited to vitamins and certain plant extracts, with a narrow range of efficacy. Therefore, there is an urgent need to identify new, safe, and effective antioxidant candidates to benefit a larger number of patients suffering from skin photodamage.

Organisms inhabiting marine environments, continuously exposed to UV radiation, have evolved a suite of protective mechanisms against phototoxicity [17], particularly cyanobacteria, which dominate marine environments and are capable of synthesizing photoprotective compounds such as mycosporine-like amino acids (MAAs), scytonemin, and carotenoids [18]. MAAs are small molecules (usually less than 400 Da) that are colorless, water-soluble, and possess high stability [19]. In addition to their UV absorption properties, substantial evidence suggests that MAAs may help prevent skin aging through various mechanisms, including antioxidant activity [20], anti-inflammatory effects [21,22], inhibition of protein glycation [23], and suppression of collagenase activity [24]. Mycosporine-glycine (M-Gly), a representative member of the mycosporine-like amino acid (MAA) family, demonstrates significant antioxidant activity. It counters lipid peroxidation triggered by peroxyl radicals and neutralizes singlet oxygen produced by endogenous photosensitizers. These properties protect marine organisms from oxidative damage caused by sunlight exposure [25,26]. Beyond its potent antioxidant properties, M-Gly has also demonstrated anti-inflammatory effects in skin cell models [22]. Nevertheless, in vivo research on M-Gly’s efficacy against skin photodamage and photoaging remain scarce. To better understand its pharmacological mechanisms, additional analytical methods are required. Metabolomics, an emerging field in systems biology, focuses on the comprehensive metabolic profiles of cells, tissues, or organisms in relation to genetic variations and external stimuli [27]. Metabolites, which are the end results of gene expression, can reveal specific metabolic patterns closely associated with physiological or pathological phenotypes [28]. Skin metabolomics elucidates the complex interactions between external stimuli and the body’s internal responses, potential metabolic biomarkers for diagnosing, prognosing, and treating both local and systemic skin diseases, while also enhancing our understanding of drug mechanisms [29]. In the past ten years, high-throughput RNA sequencing (RNA-seq) has emerged as a critical tool for examining transcriptional changes across the transcriptome [30]. Transcriptomic analysis allows for the detection of gene expression differences between healthy and diseased skin, facilitating the identification of specific genes or gene networks involved in skin diseases. Combining transcriptomics with metabolomics offers a more comprehensive biological perspective, revealing the relationship between gene expression and metabolic changes, which in turn enhances the accuracy and specificity of biomarkers.

In this study, we established a UVB-induced photodamage mouse model to evaluate the macroscopic and histopathological skin changes, as well as to quantify key antioxidants and anti-inflammatory factors in the skin. This model enabled us to assess the pharmacodynamic effects of M-Gly in alleviating UVB-induced skin damage. Ultimately, metabolomic and transcriptomic analyses were conducted to explore the mechanisms through which M-Gly ameliorates skin photodamage.

## 2. Materials and Methods

### 2.1. Reagent Materials

The RNA extraction kit (TransZol Up Plus RNA Kit, TransGen Biotech, Beijing, China), cDNA synthesis kit (TransScript^®^ One-Step gDNA Removal and cDNA Synthesis SuperMix, TransGen Biotech, Beijing, China), and TransScript^®^ II Green One-Step qRT-PCR SuperMix were purchased from TransGen Biotech Co., Ltd. (Beijing, China). 2,2′-Azinobis-(3-ethylbenzothiazoline-6-sulfonic acid) (ABTS) Free Radical Scavenging Capacity Assay Kit, SOD, CAT, malondialdehyde (MDA), glutathione (GSH), aspartate aminotransferase (AST), alanine aminotransferase (ALT) and bicinchoninic acid (BCA) assay kits were purchased from Solarbio Co., Ltd. (Beijing, China). Ascorbic acid was purchased from China National Pharmaceutical Group Corporation (Beijing, China). Matrix Metalloproteinase-1 (MMP-1), Type I procollagen, Tumor Necrosis Factor-alpha (TNF-α), Interleukin-6 (IL-6), and Interleukin-1 beta (IL-β) ELISA kits were purchased from Shanghai Enzyme-linked Biotechnology Co., Ltd. (Shanghai, China). All chemicals and solvents were analytical grade or chromatographic grade.

### 2.2. Compound Description

The compound M-Gly utilized in this study was synthesized via a recombinant biosynthetic pathway. We combined the genes mysA, mysB, and mysC, which are involved in the MAA synthesis pathway of *Microcystis aeruginosa*, and expressed them in *Escherichia coli* BL21 (DE3) to produce M-Gly (UV λmax: 310 nm; *m*/*z* 246.0972). The compound was purified using High-Performance Liquid Chromatography (HPLC, HPLC-LC2000, Hitachi, Ltd., Japan) and its structure was confirmed by UV absorption spectrum (T600, Beijing Puxi General Instrument Co., Ltd., Beijing, China), Liquid Chromatography-Mass Spectrometry/Mass Spectrometry (LC-MS/MS, Q-Exactive HF, Thermo Fisher Scientific Inc., Waltham, MA, USA), and Proton Nuclear Magnetic Resonance (1H-NMR, JNM-ECZ, JEOL Ltd., Tokyo, Japan) analysis. The HPLC chromatograms, UV absorption spectra, LC-MS/MS data, and 1H-NMR spectra are presented in the non-published material. The 1H NMR spectra of M-Gly align with the chemical shift data previously reported by Emily P. Balskus et al., 2010 [31]. Further details of this process are available in our unpublished work [32].

### 2.3. ABTS Clearance Ability Test In Vitro

The in vitro free radical scavenging capacity of M-Gly was evaluated utilizing the ABTS radical cation decolorization assay, as per the protocol provided in an ABTS assay kit. M-Gly was prepared in various concentrations (0.03125 mM, 0.0625 mM, 0.125 mM, 0.25 mM, 0.5 mM, 1 mM), and each concentration was mixed with the prepared working solution. The sample was incubated at room temperature for a duration of 6 min in a light-protected environment, followed by the quantification of absorbance at a wavelength of 405 nm. Subsequently, the ABTS scavenging rate was determined. As a water-soluble compound, M-Gly was compared to ascorbic acid, another water-soluble antioxidant, which was used as the positive control in this study.

### 2.4. Animal Grouping and Treatment

All subsequent animal studies were performed in compliance with the ethical guidelines granted by the Animal Ethics Committee of Qingdao University (Certificate No. 20240308ICR6420240418050). Thirty ICR female mice, approximately 4 weeks old, were purchased from Jinan Pengyue Co., Ltd. Following a one-week acclimatization period, they were randomly allocated into four groups. The dorsal fur of the mice was shaved using a depilatory cream, and the following day, they were exposed to UVB lamps (Nanjing Huaqiang Electronics Co., Ltd., Nanjing, China). The UVB exposure lasted for 40 min each day, delivering a dose of 120 mJ/cm^2^ over a continuous period of 30 days. The specific groupings are listed in Table 1. The normal control group (NC, *n* = 6) received no additional treatment. For the model negative control group (MC, *n* = 8), intraperitoneally injected 0.9% normal saline without M-Gly followed by UVB irradiation. For the high-dose experimental group (H-MG, *n* = 8), 0.2 mL of 2 mg/kg M-Gly was intraperitoneally injected followed by UVB irradiation. For the low-dose experimental group (L-MG, *n* = 8), 0.2 mL of 0.2 mg/kg M-Gly was intraperitoneally injected followed by UVB irradiation. Prior to each injection experiment, the freeze-dried M-Gly powder was reconstituted in normal saline. A 0.22-μm filter membrane was employed to sterilize the solution by removing bacteria, and injections were administered every two days. After 30 days, photographic documentation and skin tissue sampling were performed on the mice. Throughout the experiment, their diet was monitored daily, and body weight was recorded weekly. Skin tissues were rapidly frozen in liquid nitrogen and stored at a temperature of −80 °C for subsequent analysis, or fixed in 4% paraformaldehyde (Sinopharm Chemical Reagent Co., Ltd., Beijing, China) for embedding and sectioning.

### 2.5. Biosafety Assessment

To assess potential toxic side effects, routine blood tests (three categories) were conducted on mice in the intraperitoneal injection group. Wuhan Servicebio Technology Co., Ltd. (Wuhan, China) was responsible for the testing, utilizing a Mindray automatic veterinary blood cell analyzer (BC-2800vet, Shenzhen Mindray Animal Medical Technology Co., Ltd., Shenzhen, China). Additionally, serum levels of AST and ALT were measured. Histological assessment using Hematoxylin and Eosin (H&E, Merck KGaA, Darmstadt, Germany.) staining was performed on the liver, spleen, and kidney to observe any organ damage in the mice.

### 2.6. Histopathology

Histological evaluation of skin morphology was conducted utilizing H&E staining. Collagen fibers were analyzed using Masson’s trichrome staining, applying iron hematoxylin (Wuhan Servicebio Biotechnology Co., Ltd., Wuhan, China), ponceau acid fuchsin-phosphomolybdic acid (Wuhan Servicebio Biotechnology Co., Ltd., Wuhan, China), and aniline blue (Wuhan Servicebio Biotechnology Co., Ltd., Wuhan, China). Mast cell distribution in skin tissue was observed through toluidine blue staining. All stained sections were examined under an optical microscope, with quantitative analysis conducted using ImageJ software (version 1.54g) for accurate measurements and comparisons.

### 2.7. Enzyme-Linked Immunosorbent Assay (ELISA)

Mouse skin tissue samples, each weighing 0.1 g, were homogenized in 1 mL of physiological saline. The resulting supernatant was then collected for further analysis. Total protein was extracted from the homogenate, followed by quantification using a BCA assay kit. Levels of MMP-1, type I procollagen, TNF-α, IL-6, and IL-1β in the supernatant were determined employing ELISA, strictly adhering to the instructions provided by the manufacturer.

### 2.8. Assessment of Oxidative Damage Indicators in Mice Skin

Skin tissue samples were weighed and subsequently homogenized using commercial extraction reagents at a proportion of 1 mL of reagent per 0.1 g of tissue. After homogenization, the mixture was centrifuged at 8000× *g* for 10 min at 4 °C, and the supernatant was collected. The levels of SOD, CAT, GSH, and MDA in the supernatant were then quantified.

### 2.9. RNA Extraction and Skin Transcriptome Analysis

Total RNA was extracted from skin tissue using TRIzol^®^ Reagent (TransGen Biotech, Beijing, China) and assessed for quality with the Agilent 5300 Bioanalyzer (Agilent Technologies, Inc, Santa Clara, CA, USA) and NanoDrop ND-2000 (Thermo Fisher Scientific Inc., MA, USA). High-quality RNA, meeting specific criteria (OD260/280: 1.8–2.2, OD260/230 ≥ 2.0, RQN ≥ 6.5, 28S:18S ≥ 1.0, and yield ≥ 1 μg), was selected for library construction. RNA purification, reverse transcription, library construction, and sequencing were conducted at Shanghai Majorbio Bio-pharm Biotechnology Co., Ltd. (Shanghai, China) on the NovaSeq X Plus platform (PE150, Illumina, Inc., San Diego, CA, USA). Transcript expression levels were calculated using TPM, and gene abundance was quantified with RSEM. Differential expression analysis was performed using DESeq2 or DEGseq, with significant DEGs defined as those with |log2FC| ≥ 1 and FDR < 0.05 (DESeq2) or FDR < 0.001 (DEGseq). Functional enrichment analyses, including Gene Ontology (GO) and Kyoto Encyclopedia of Genes and Genomes (KEGG) pathway analyses, were conducted with Bonferroni-corrected *p*-values < 0.05 using Goatools (released in 2015) and SciPy (Version1.0.0).

### 2.10. Untargeted Skin Metabolome Analysis

A 50 mg sample of skin tissue was weighed for metabolite extraction using 400 μL of a methanol-water solution (4:1 *v*/*v*) containing 0.02 mg/mL L-2-chlorophenylalanine as an internal standard. The mixture was incubated at −10 °C, disrupted using a High Throughput Tissue Crusher (50 Hz for 6 min), and ultrasonicated at 40 kHz for 30 min at 5 °C. After protein precipitation at −20 °C, the samples were centrifuged at 13,000× *g* for 15 min at 4 °C. The supernatant was collected for liquid chromatography-tandem mass spectrometry (LC-MS/MS) analysis on a UHPLC-Q Exactive system (Q Exactive HF, Thermo Fisher Scientific Inc., MA, USA). A 2 μL sample was separated on an HSS T3 column (100 mm × 2.1 mm, 1.8 μm), with a solvent gradient program consisting of solvent A (0.1% formic acid in water) and solvent B (0.1% formic acid in acetonitrile: isopropanol). The injection volume was 2 μL, with a flow rate of 0.4 mL/min and column temperature maintained at 40 °C. All samples were stored at 4 °C during analysis.

Mass spectrometric data were acquired using a Thermo UHPLC-Q Exactive Mass Spectrometer (Q Exactive HF, Thermo Fisher Scientific Inc., MA, USA) with an electrospray ionization (ESI) source, operating in both positive and negative ion modes. The optimized parameters for the mass spectrometer were as follows: heater temperature at 400 °C, capillary temperature at 320 °C, sheath gas flow rate at 40 arbitrary units (arb), and auxiliary gas flow rate at 10 arb. The ion-spray voltage was set to −2800 V for negative mode and 3500 V for positive mode. The normalized collision energy was ramped from 20 V to 40 V to 60 V for MS/MS acquisition. The full MS resolution was set to 70,000, while the MS/MS resolution was set to 17,500. Data acquisition was conducted in Data Dependent Acquisition (DDA) mode, with a mass range of 70 to 1050 *m*/*z*.

Raw LC/MS data were processed using Progenesis QI software (version 2.0, Waters Corporation, Milford, CT, USA), and metabolites were identified via HMDB, Metlin, and Majorbio databases. The processed data were then uploaded to the Majorbio cloud platform (https://cloud.majorbio.com, accessed on 18 October 2024) for further analysis. Variance analysis, Principal component analysis (PCA), and orthogonal partial least squares discriminant analysis (OPLS-DA) was performed using the R packages (Version 1.6.2), with 7-cycle validation. Additionally, Student’s *t*-test and fold change analysis were applied. Metabolites with a VIP score > 1 and *p*-value < 0.05 were considered significantly different. KEGG pathway enrichment was analyzed with Goatools (released in 2015) and KOBAS 2.1.1, and integrated transcriptome-metabolome analysis was conducted using iPath 3.0.

### 2.11. Quantitative Real-Time PCR

Total RNA from mouse skin was reverse transcribed using the TransScript^®^ One-Step gDNA Removal (TransGen Biotech, Beijing, China) and cDNA Synthesis SuperMix (TransGen Biotech, Beijing, China). The reaction was incubated at 42 °C for 15 min and then heated at 85 °C for 5 s. mRNA expression was measured by Quantitative Real-time PCR (qRT-PCR) using a LightCycler^®^96 SW 1.1 instrument (Roche, Basel, Switzerland). The 20 µL qPCR reaction consisted of 2 µL of cDNA template, 0.4 µL of forward primer, 0.4 µL of reverse primer, 10 µL of 2×PerfectStart^®^ Green qPCR SuperMix (TransGen Biotech, Beijing, China), and 7.2 µL of nuclease-free water. The PCR cycling conditions were as follows: 30 s at 94 °C, followed by 45 cycles of 5 s at 94 °C, 15 s at 55 °C, and 10 s at 72 °C. Relative gene expression was calculated using the 2^−∆∆CT^ method [33]. In this experiment, the sequences for the forward and reverse primers of the *Nrf2* gene are AGCACAGCCAGCACATTCTCC and GACCAGGACTCACGGGAACTTC, respectively. Similarly, the sequences for the forward and reverse primers of the *HO-1* gene are AAGACCGCCTTCCTGCTCAAC and TCTGACGAAGTGACGCCATCTG, respectively.

### 2.12. Statistical Analysis

Data analysis was performed using IBM SPSS version 22.0 (Chicago, IL, USA) and GraphPad Prism 9.0 (La Jolla, CA, USA). We utilized a one-way analysis of variance (ANOVA), supplemented by the least significant difference (LSD) test, to compare the means across the experimental groups.

## 3. Results

### 3.1. ABTS Free Radicals Scavenging Capacity of M-Gly

**Figure 1:**
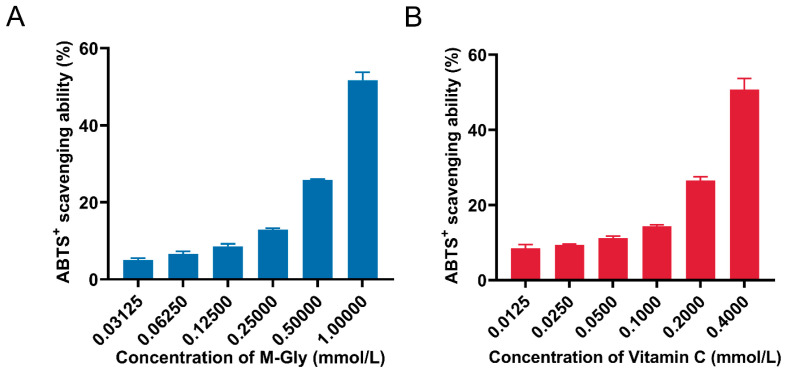
ABTS radical scavenging capacity of M-Gly (**A**) and vitamin C (**B**).

ABTS^+^ is a water-soluble cationic free radical, and the ABTS method is extensively used for assessing the free radical scavenging capacity in both water-soluble and fat-soluble samples. The free radical scavenging activities of M-Gly aqueous solutions at varying concentrations were determined by their ability to decolorize ABTS^+^ in vitro. The results are presented in Figure 1. Figure 1A illustrates the ABTS^+^ scavenging capacity of M-Gly at different concentrations (0.03125 mM, 0.0625 mM, 0.125 mM, 0.25 mM, 0.5 mM, and 1 mM), while Figure 1B depicts the same for vitamin C (VC) at varying concentrations (0.0125 mM, 0.025 mM, 0.05 mM, 0.1 mM, 0.2 mM, and 0.4 mM). It is observed that the ABTS^+^ scavenging capacity of M-Gly increases with concentration, with a median maximum inhibitory concentration (IC50) of 2.258 mM, compared to an IC50 value of 0.503 mM for VC.

### 3.2. Body Weight and Diet of Mice During the Experiment

**Figure 2:**
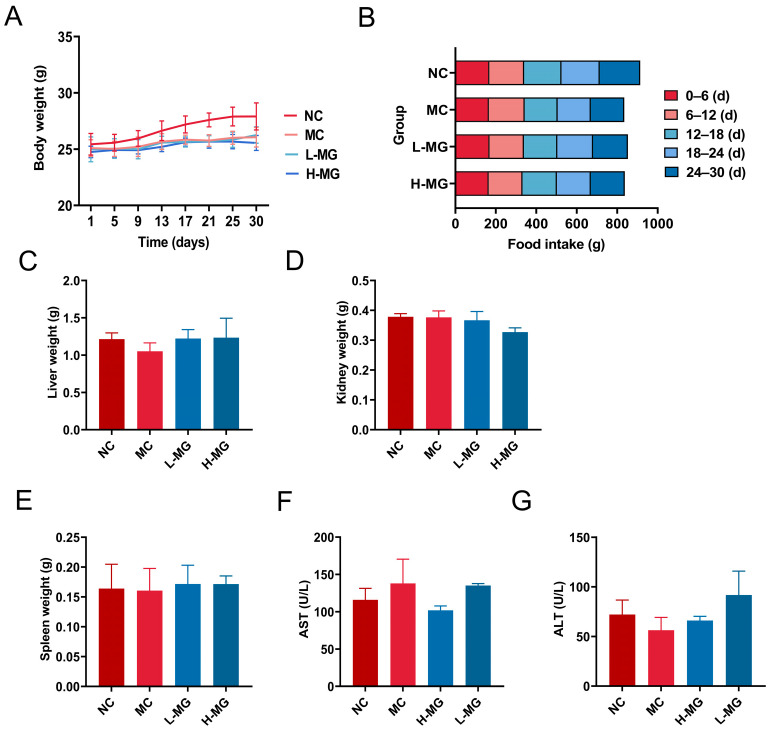
The body weight and food intake records of mice during the modeling process and the results of M-Gly biosafety detection of mice. (**A**) Line chart of body weight change in mice. (**B**) The diet condition of mice. (**C**) Liver weight was recorded in each group. (**D**) Kidney weight was recorded in each group. (**E**) Spleen weight was recorded in each group. (**F**) Serum ALT levels were detected in each group. (**G**) Serum AST levels were detected in each group. NC represents the normal control group, MC represents the model negative group, H-MG represents the high-dose experimental group, and L-MG represents the low-dose experimental group.

The weight changes in mice and the statistical results of their food intake across different groups are depicted in Figure 2A,B. Observations revealed that the body weight of mice in all groups gradually increased, with no significant decrease. The total food intake of the mice did not differ significantly from that of the control group. These results suggest that, aside from the skin damage caused by UVB irradiation, the overall health of the mice remained good during the experiment.

### 3.3. The Results of Biosafety Assessment

#### 3.3.1. Blood Routine Test of Mice

The hematological parameters of mice in each group are presented in Table 2. For various hematological parameters including white blood cell count, lymphocyte count, monocyte count, neutrophil count, lymphocyte percentage, monocyte percentage, neutrophil percentage, red blood cell count, hemoglobin concentration, hematocrit, mean corpuscular volume, mean corpuscular hemoglobin, mean corpuscular hemoglobin concentration, red cell distribution width, platelet count, mean platelet volume and platelet distribution width, no significant differences were observed between the treatment and control groups (*p* < 0.05). This indicates that M-Gly did not adversely affect the routine blood parameters in mice.

#### 3.3.2. Effects of M-Gly Intervention on Major Organs of Mice

**Figure 3:**
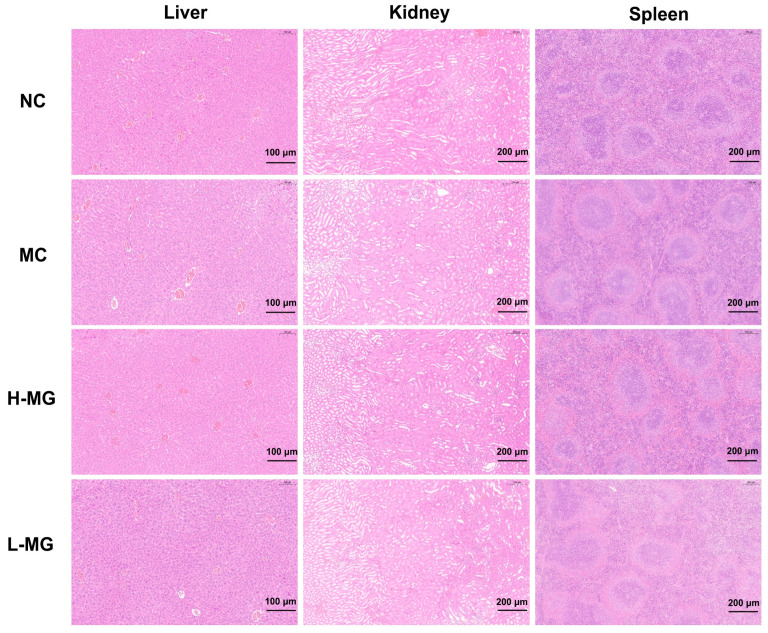
Hematoxylin and eosin (H&E) staining section images of the liver, kidney, and spleen of mice in each group. NC represents the normal control group, MC represents the model negative group, H-MG represents the high-dose experimental group, and L-MG represents the low-dose experimental group.

To further assess the biocompatibility of M-Gly in mice, we recorded the weights of the liver, kidney, and spleen from each group on the day of sampling, with results presented in Figure 2C–E. There were no notable differences in the organ assessments among the groups. Additionally, we measured the levels of AST and ALT in serum to evaluate the impact of M-Gly on liver function. As depicted in Figure 2F,G, there was no significant difference in serum AST and ALT levels between the M-Gly treated group and the control group, suggesting that M-Gly did not significantly affect liver function. Histopathological analysis was conducted on the three major organs (liver, kidney, and spleen), with H&E staining images displayed in Figure 3. Compared to untreated and saline-treated mice, intraperitoneal injection of M-Gly did not induce pathological changes in these organs, indicating no adverse effects on the liver, kidney, and spleen of the mice.

### 3.4. Analysis of Macroscopic Appearance and Histopathologic Features of Mice Skin

**Figure 4:**
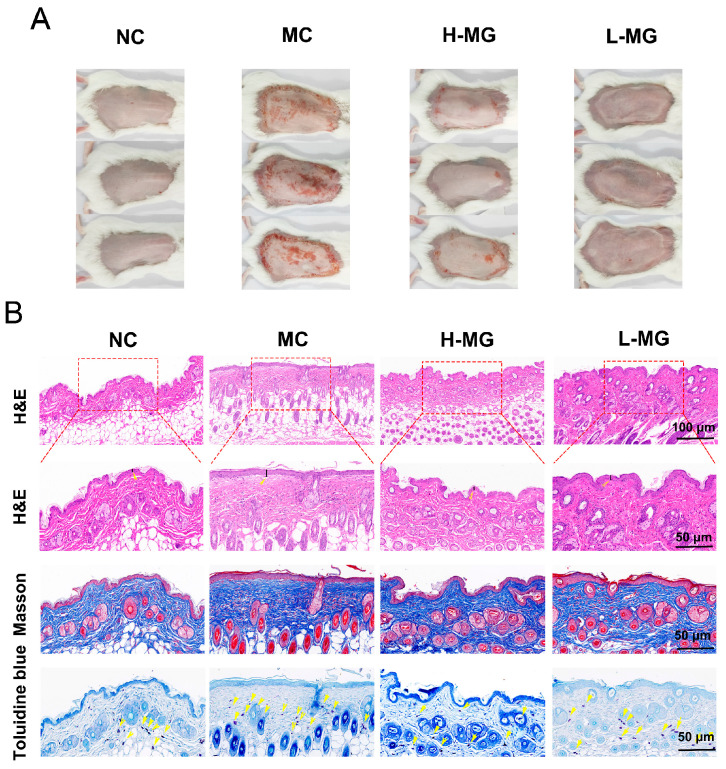
The preventive and therapeutic effects of M-Gly administration on UVB-induced skin damage in mice. (**A**) Images of the back skin of mice, *n* = 6 or *n* = 8. (**B**) Histological staining of mouse dorsal skin (H&E, Masson staining, and Toluidine blue staining), *n* = 3. In the Masson staining results, blue represents collagen fibers.

**Figure 5:**
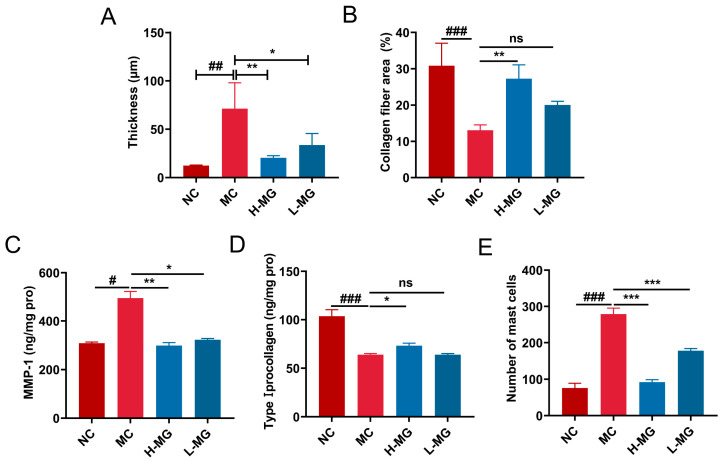
Indices of skin damage induced by UVB radiation in mice. (**A**) Statistical analysis of the skin thickness. (**B**) Statistical analysis of the density of collagen fibers in the dermis of mouse dorsal skin. (**C**) MMP-1 levels in mice skin. (**D**) Type I procollagen levels in mice skin. (**E**) Statistical analysis of the mast cells number in the mouse dorsal skin. NC represents the normal control group, MC represents the model negative group, H-MG represents the high-dose experimental group, and L-MG represents the low-dose experimental group. # *p* < 0.05, ## *p* < 0.01, ### *p* < 0.001, compared with the NC group; * *p* < 0.05, ** *p* < 0.01, *** *p* < 0.001, compared with the MC group; ns represents no statistically significant difference between the two groups.

Figure 4A illustrates the dorsal skin morphology of mice. Mice subjected to UVB radiation displayed erythema and wrinkles, in contrast to the normal group. However, those treated with M-Gly exhibited significantly reduced skin irritation compared to the model group. To further assess the impact of intraperitoneal M-Gly on UVB-induced skin damage, we conducted histological staining on tissue sections, including H&E, Masson’s trichrome, and toluidine blue staining. Based on the results of H&E staining (Figure 4B) and the statistical analysis (Figure 5A), compared to the skin of normal mice, the thickness of the epidermal layer in the dorsal skin of mice subjected to long-term UVB irradiation was significantly increased (*p* < 0.05). Intervention with M-Gly markedly reduced the epidermal thickness in mice (*p* < 0.01, *p* < 0.05). Based on the results of Masson’s trichrome staining (Figure 4B), the dermis of the normal group mice contained uniformly distributed, wavy fiber tissues. Following UVB exposure, the dermal collagen fibers became disordered, with broken and loose structures. Some fibers aligned parallel to the skin surface, losing their wavy pattern, and the density of collagen fibers was significantly reduced (Figure 5B, *p* < 0.001). M-Gly administration ameliorated the structural damage to collagen fibers in the dermis, effectively increasing fiber density in the high-dose group (Figure 5B, *p* < 0.01), and facilitating the reconstruction of damaged tissue. Moreover, M-Gly treatment significantly decreased MMP-1 levels in the skin of UVB-irradiated mice (Figure 5C, *p* < 0.001), indicating protection against UVB-induced collagen degradation. Concurrently, M-Gly significantly elevated type I procollagen levels compared to the UVB-only group (Figure 5D, *p* < 0.001 or *p* < 0.01), enhancing skin repair and collagen preservation. Toluidine blue staining revealed mast cell infiltration (Figure 4B), with quantification shown in Figure 5E. The model group exhibited increased mast cell recruitment in the dermis compared to the normal group, suggesting UVB-induced mast cell activation. M-Gly treatment significantly reduced mast cell numbers in the skin (*p* < 0.001), indicating a decrease in skin inflammation.

### 3.5. M-Gly Injection Alleviated UVB-Induced Oxidative Stress in Mice Skin

**Figure 6:**
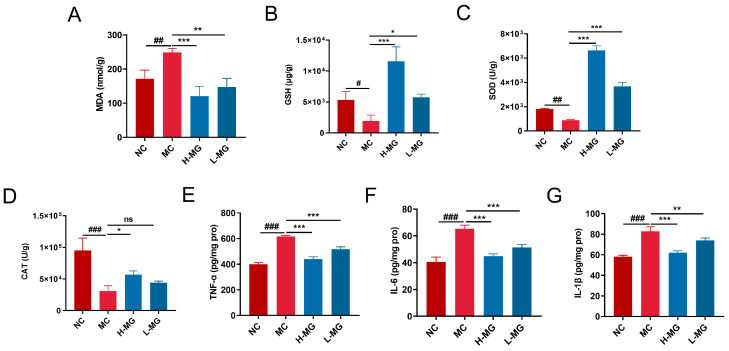
(**A**–**D**) Effects of M-Gly on levels of MDA, SOD, CAT, and GSH in UVB damaged skin of mice. (**E**–**G**) Effects of M-Gly on the inflammatory factors (TNF-α, IL-6, and IL-1β) in UVB damaged skin of mice. NC represents the normal control group, MC represents the model negative group, H-MG represents the high-dose experimental group, and L-MG represents the low-dose experimental group. # *p* < 0.05, ## *p* < 0.01, ### *p* < 0.001, compared with the NC group; * *p* < 0.05, ** *p* < 0.01 and *** *p* < 0.001, compared with the MC group; ns represents no statistically significant difference between the two groups.

The MDA content in the dorsal skin tissue of mice was measured after a 30-day UVB exposure, as depicted in Figure 6A. The MDA levels in the dorsal skin significantly increased due to UVB radiation compared to the control group (*p* < 0.01). However, intraperitoneal injection of M-Gly markedly reduced the elevated MDA levels post-UVB exposure (*p* < 0.001, *p* < 0.01). The activity levels of endogenous antioxidants, SOD and CAT, in the dorsal skin, were also assessed (Figure 6B,C). UVB radiation significantly reduced the activities of both SOD and CAT (*p* < 0.01, *p* < 0.001). M-Gly intervention restored these enzyme activities in the skin tissue. Compared to the model group, both high and low doses of M-Gly significantly up-regulated SOD activity. High dose M-Gly significantly up-regulated CAT activity (*p* < 0.05), while the low dose had no significant effect. Figure 6D presents the GSH content measurements. UVB radiation significantly decreased GSH levels in the dorsal skin tissue of the mice (*p* < 0.05). In contrast, M-Gly intervention significantly increased GSH levels (*p* < 0.001, *p* < 0.01).

### 3.6. M-Gly Injection Alleviated UVB-Induced Inflammatory Response in Mice Skin

Inflammatory cytokine levels in the skin of mice were assessed using ELISA assays. As shown in Figure 6E–G, the levels of TNF-α, IL-6, and IL-1β were markedly elevated in the model group relative to the normal control (NC) group (*p* < 0.001), indicating that UVB irradiation provoked skin inflammation. In contrast, M-Gly treatment significantly decreased the expression of these cytokines (*p* < 0.001, *p* < 0.01), suggesting its potential anti-inflammatory properties.

### 3.7. Effects of M-Gly Injection on Mice Skin Metabolic Profiles

**Figure 7:**
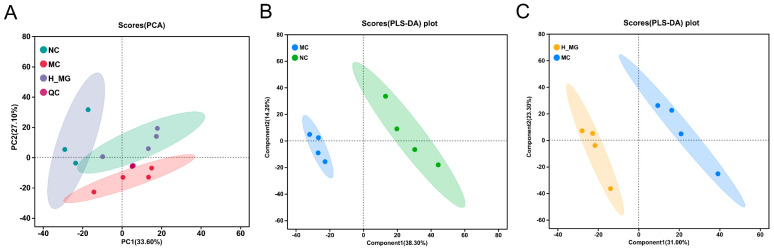
The PCA and PLS-DA analysis results of the samples from each group in the skin metabolomics. (**A**) PCA score plot of NC group, MC group, M-Gly group, and QC samples. (**B**) PLS-DA score plot of NC group vs. MC group. (**C**) PLS-DA score plot of MC group vs. M-Gly group. The confidence ellipse indicates that the set of “real” samples is distributed in this region with 95% confidence.

**Figure 8:**
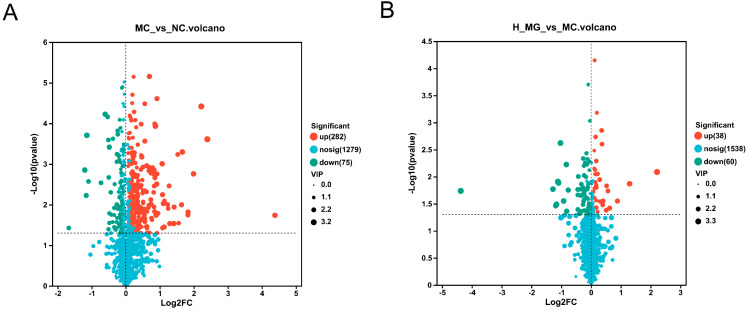
The volcano plot results of metabolites in different experimental groups. (**A**) Volcano plot of significant differential metabolites of NC group vs. MC group. (**B**) Volcano plot of significant differential metabolites of MC group vs. M-Gly group.

**Figure 9:**
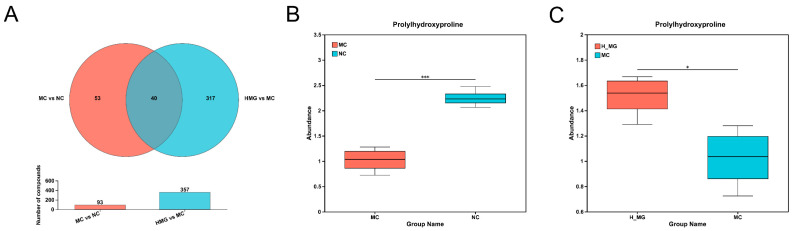
Specific differential metabolite analysis of M-Gly intervention. (**A**) Venn diagram of differential metabolites between the two comparison groups (NC vs. MC and MC vs. M-Gly). (**B**) The content of prolylhydroxyproline in skin metabolites between NC group and MC group. (**C**) The content of prolylhydroxyproline in skin metabolites between the MC group and M-Gly group. * Represents *p* < 0.05, *** represents *p* < 0.001.

**Figure 10:**
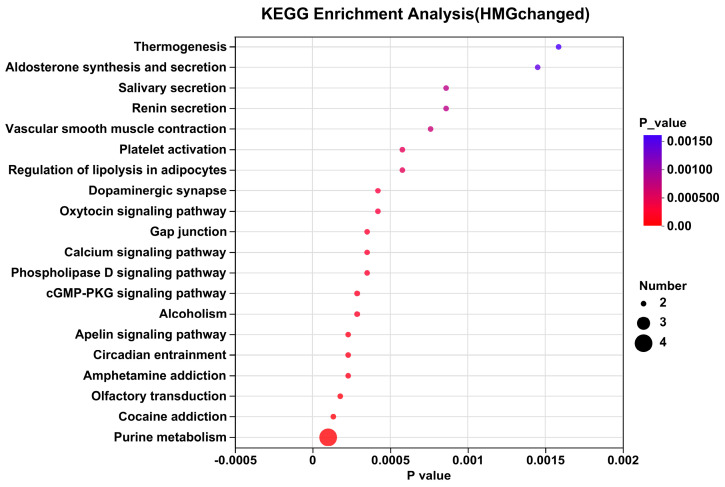
Metabolic pathway enrichment analysis of identified differential metabolites. The size of the bubbles in the figure represents the number of metabolites enriched in the pathway, while the color of the bubbles indicates the significance level of the enrichment, as represented by the *p*-value.

UPLC-MS/MS was utilized to analyze skin metabolite variations between the MC and NC groups, as well as between the M-Gly and MC groups. Through this analysis, a total of 410 annotated metabolites were identified, including ions detected in both ESI+ and ESI− modes. In this study, PCA and PLS-DA methods were applied to analyze differences in skin metabolism among UVB-damaged mice following M-Gly intervention, identifying potential differential metabolites. PCA score plot in Figure 7A shows sample clustering and group separation, with distinct metabolic profiles among groups and minimal intra-group variation, suggesting UVB’s significant impact on mouse skin metabolism and M-Gly’s regulatory influence. The PLS-DA score plot (Figure 7B,C) highlights the model’s classification efficacy, indicating significant metabolic profile differences. The MC group had 282 upregulated and 75 downregulated biomarkers compared to the NC group, while the M-Gly group exhibited 38 upregulated and 60 downregulated biomarkers relative to the MC group (*p* < 0.05). Volcano plots in Figure 8A,B summarize these differential metabolites. The Venn analysis identified 40 shared differential metabolites between the two groups, as illustrated in Figure 9A. Notably, the levels of prolylhydroxyproline (Pro-Hyp), a key metabolite in skin collagen synthesis, were significantly reduced by UVB treatment (*p* < 0.001). In contrast, the M-Gly treatment led to a significant increase in Pro-Hyp levels (*p* < 0.05). The changes in Pro-Hyp content are illustrated in Figure 9B,C. Figure 10 presents the results of the KEGG pathway enrichment analysis for the differential metabolites, highlighting several highly enriched pathways. Key pathways of interest are the metabolism of amino sugars and nucleotide sugars, linoleic acid metabolism, carbohydrate digestion and absorption, sphingolipid signaling pathways, and choline metabolism in cancer.

### 3.8. Transcriptomics Analysis of Mice Skin Tissue

**Figure 11:**
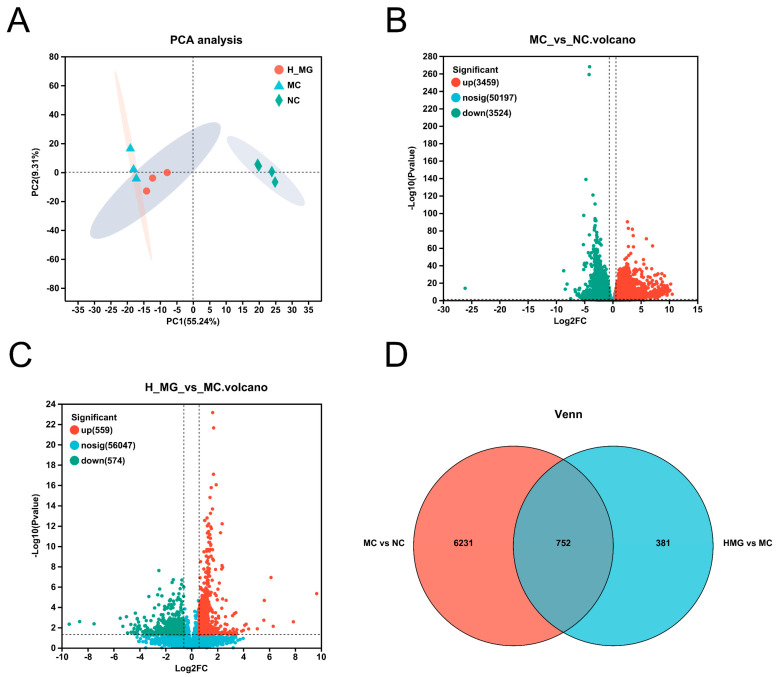
Effects of M-Gly intervention on skin transcriptome. (**A**) PCA score plot of NC group, MC group, and M-Gly group samples. The confidence ellipse indicates that the set of "real" samples is distributed in this region with 95% confidence. (**B**) Volcano plot of significant differential genes (DEGs) of MC group vs. NC group. (**C**) Volcano plot of significant DEGs of M-Gly group vs. MC group. (**D**) Venn diagram of DEGs between the two comparison groups (NC vs. MC and MC vs. M-Gly).

According to PCA (Figure 11A), samples from the NC group (green diamonds), MC group (blue triangles), and H-MG group (orange circles) clustered distinctly, indicating different gene expression profiles and lower intra-group variability. Based on the criteria of false discovery rate (FDR) (fold change > 1.5, *p* < 0.05), UVB irradiation resulted in significant downregulation of 3524 genes and upregulation of 3459 genes, as compared to the gene expression levels in normal mouse skin. This is evident in the volcano plot (Figure 11B). However, compared to UVB treatment alone, M-Gly intervention significantly downregulated the expression of 574 genes and upregulated 559 genes, as shown in Figure 11C. According to Venn analysis (Figure 11D), among the 6231 genes that exhibited significant changes due to UVB treatment, M-Gly intervention restored the expression of 752 genes. These 752 differentially expressed genes (DEGs) were then compiled into a gene set, denoted as H_MG_MC.

**Figure 12:**
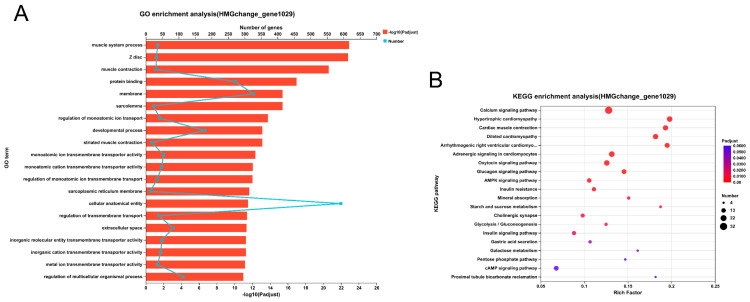
Functional enrichment analysis of target genes. (**A**) GO enrichment analysis of identified DEGs. (**B**) KEGG enrichment analysis of identified DEGs.

GO and KEGG analyses were conducted to functionally enrich the overlapping DEGs identified previously. The GO enrichment analysis revealed significant enrichment of these DEGs in several pathways, as depicted in Figure 12A. These included pathways related to the regulation of monoatomic ion transport, developmental processes, regulation of multicellular organismal processes, regulation of localization, response to stimulus, biological regulation, regulation of system processes, inorganic molecular entity transmembrane transporter activity, metal ion transmembrane transporter activity, cytoskeletal protein binding, and signaling receptor binding. In the KEGG enrichment analysis (Figure 12B), it was found that these DEGs are significantly enriched in pathways such as the calcium signaling pathway, AMPK signaling pathway, starch, and sucrose metabolism, conjoint analysis of transcriptome and metabolome, nitrogen metabolism, alanine, aspartate, and glutamate metabolism, histidine metabolism, ether lipid metabolism, sphingolipid metabolism, and D-amino acid metabolism.

**Figure 13:**
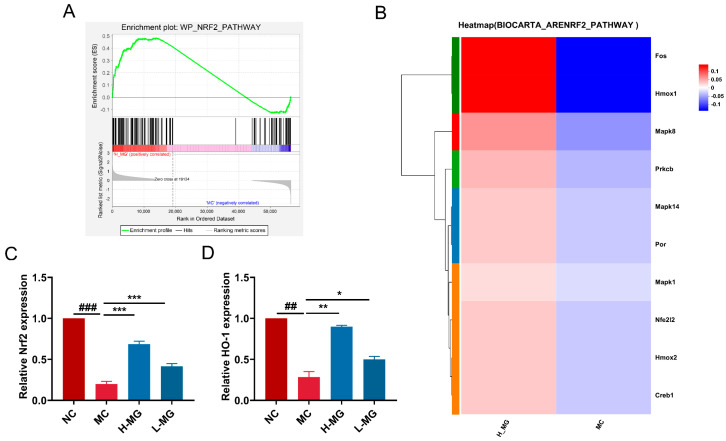
Effect of M-Gly intervention on Nrf2/HO-1 pathway. (**A**) GSEA enrichment plot of the WP_NRF2_PATHWAY gene set in the M-Gly group and MC group. (**B**) The expression heatmap of genes involved in the GSEA. Red represents a higher expression level of the gene in the sample, while blue represents a lower expression level. (**C**) The expression level of Nrf2 mRNA. (**D**) The expression level of HO-1 mRNA. NC represents the normal control group, MC represents the model negative group, H-MG represents the high-dose experimental group, and L-MG represents the low-dose experimental group. ## *p* < 0.01, ### *p* < 0.001, compared with the NC group; * *p* < 0.05, ** *p* < 0.01 and *** *p* < 0.001, compared with the MC group.

Gene Set Enrichment Analysis (GSEA) was performed on the oxidative stress-related genes within the H_MG_MC gene set. The GSEA enrichment plot (Figure 13A) indicated significant expression differences in the WP_NRF2_PATHWAY gene set from the MSigDB database between the H-MG and MC groups. The majority of genes in this pathway showed a strong positive correlation with the skin phenotype of H-MG group mice compared to the model group, with a Padjust value less than 0.25 and a Normalized Enrichment Score (NES) of 1.186. The leading-edge genes, which contributed the most to the enrichment score, included MAPK1, CREB1, HMOX2, PRKCB, NFE2L2, POR, MAPK14, HMOX1, FOS, and MAPK8. Figure 13B presents the results of a clustering analysis on the set of DEGs, illustrating the expression trends of the target genes across different groups. The heatmap reveals significant mRNA expression differences between the model group and the M-Gly intervention group in mice. The gene set exhibits a trend of higher expression in the M-Gly group, suggesting that M-Gly may restore the UVB-induced redox imbalance by activating the antioxidant pathway. The heatmap colors represent normalized gene expression levels, ranging from high (red) to low (blue). qRT-PCR was used to measure the mRNA expression levels of the core transcription factor NFE2L2 (Nrf2) and its downstream antioxidase HMOX1 (HO-1) in the skin of mice across different groups (Figure 13C,D). Compared with the NC group, the mRNA expression levels of Nrf2 and HO-1 in the MC group were both significantly decreased (*p* < 0.001 and *p* < 0.01, respectively). In contrast, the mRNA levels of both Nrf2 and HO-1 were significantly elevated in the H-MG and L-MG groups compared to the MC group (*p* < 0.001, *p* < 0.01, and *p* < 0.05).

## 4. Discussion

Excessive ROS can lead to oxidative damage in the skin by directly damaging lipids, proteins, and DNA. This oxidative damage activates oncogenes, degrades collagen, and increases the permeability of cell membranes [33]. Serving as the primary protective barrier, the skin is continually subjected to ROS from both endogenous and exogenous sources. The balance between the antioxidant system and ROS is critical for maintaining skin health. UV exposure induces the excessive production of ROS, disrupting this balance. This leads to lipid peroxidation of cell membranes, inhibits the activity of antioxidant enzymes such as SOD and CAT, and reduces the levels of antioxidant peptides like reduced GSH [34,35]. These changes result in cellular structural and functional impairments, which can ultimately lead to cell apoptosis [36]. MDA, a terminal product of lipid peroxidation, is commonly used as a biomarker for oxidative stress. The levels of CAT, SOD, and GSH are indicative of the body’s capacity to detoxify ROS. Therefore, the use of antioxidants is an effective strategy for UV skin protection, as they neutralize intracellular free radicals, reduce oxidative stress, and thereby mitigate oxidative damage to skin cells.

M-Gly is a water-soluble, small-molecule antioxidant. Using the ABTS assay, we confirmed that M-Gly exhibits effective free radical scavenging capacity in vitro. Additionally, previous studies employing density functional theory (DFT) demonstrated that M-Gly has metal-chelating abilities, allowing it to bind with metal ions such as Fe and Cu [37]. This suggests that M-Gly may reduce oxidative damage and delay skin aging by inhibiting free radical reactions catalyzed by metal ions. We therefore propose that M-Gly, which functions as a natural antioxidant in marine organisms, has the potential to serve as an active ingredient for UV-protective skin care. However, reports on the in vivo activity of M-Gly remain scarce. In this study, we investigated the efficacy and molecular mechanisms of M-Gly in alleviating UV-induced photodamage in an ICR mouse model. Administration of M-Gly did not exert a significant influence on body weight, blood counts, or organ indices of the mice, and preserved normal morphology of the liver, kidneys, and spleen without inducing hepatotoxicity. We therefore consider the M-Gly doses used in this study (0.2 mg/kg and 2 mg/kg) to be within a safe range. After UVB irradiation, MDA levels in mouse skin were significantly elevated, while CAT, SOD, and GSH levels were markedly reduced, indicating the effective establishment of the photodamage model. Compared to the UVB group, M-Gly intervention reduced MDA levels and increased CAT, SOD, and GSH levels in a dose-dependent manner. These findings suggest that M-Gly helps maintain redox balance in skin cells by alleviating UV-induced oxidative stress in mouse skin. Collagen serves as the predominant structural protein within human skin, responsible for its tensile strength. Mature dermal collagen originates from precursor molecules known as procollagen [38]. Matrix metalloproteinases (MMPs) are transmembrane zinc-dependent endopeptidases that degrade collagen and elastin fibers in the skin [39]. UV exposure and the release of reactive oxygen species pathologically upregulate MMP activity, leading to collagen depletion and resulting in skin aging signs such as sagging and wrinkles [24]. Through histopathological analysis of mouse dorsal skin, we found that M-Gly alleviates UVB-induced collagen depletion in the dermis, increases collagen deposition, and reverses structural damage. M-Gly also upregulated type I procollagen expression and reduced MMP-1 level in photoaged skin, demonstrating a protective effect during the process of UV-induced skin aging. These results align with those reported in the study conducted by Ryu et al. [21], which reported a concentration-dependent inhibitory effect of UV-A radiation on MMPs, such as MMP-1 and elastase. They further noted that at a concentration of 40μM, porphyra-334 could enhance the synthesis of procollagen in the skin. A previous study found that M-Gly exerts a strong inhibitory effect on collagenase in vitro [40]. It is hypothesized that the metal chelation activity of M-Gly, potentially mediated by its interaction with iron and calcium ions, may play a significant role in this effect [23,41]. However, the exact mechanism underlying this activity remains unclear, and further experiments are needed to elucidate it. In fact, skin wounds typically exhibit elevated levels of MMPs, and excessive protease activity is considered a major factor that impairs healing by degrading newly formed tissue and growth factors [42]. Based on the above results, it can be concluded that M-Gly has the potential to promote wound healing by inhibiting MMP activity. Similarly, metal nanoparticles (gold, silver, zinc) have been extensively studied in wound healing, particularly silver nanoparticles, which not only exhibit antimicrobial activity but also inhibit MMP activity, thereby improving epithelial formation and promoting collagen fiber deposition [42,43,44].

Prolonged UVB exposure can also lead to an increase in the number of mast cells in the dermis, triggering an inflammatory response in the skin [45]. In this investigation, toluidine blue staining was employed to detect mast cells within mouse skin. Observation of the tissue sections revealed a significant increase in mast cell numbers within the dermis of the UVB group, while M-Gly intervention markedly reversed this effect. This finding is consistent with the study by Xianrong Zhou et al. [46], which demonstrated that intraperitoneal injection of β-nicotinamide mononucleotide mitigated photodamage in mice. Additionally, the expression levels of IL-6, TNF-α, and IL-1β were elevated following UVB exposure, indicating a marked imbalance in inflammatory cytokines. After M-Gly intervention, this cytokine imbalance was reversed in a dose-dependent manner, further demonstrating that M-Gly can mitigate the inflammatory response induced by UVB exposure.

To elucidate the mechanisms behind M-Gly’s therapeutic effects on UV-induced skin damage, we employed transcriptomic and metabolomic technologies. Our findings indicate that the intraperitoneal administration of M-Gly exerts regulatory effects on both the metabolic processes and the gene expression networks within the skin. We initially employed UHPLC-MS/MS to analyze the metabolites in the exposed dorsal skin of mice across different groups. The results revealed significant differences in skin metabolites between the normal control group, the UVB model group, and the M-Gly intervention group. A total of 34 distinct metabolic markers were identified, among which Pro-Hyp caught our attention. Pro-Hyp is a naturally occurring dipeptide in the skin, particularly within the structure of collagen [47,48]. This dipeptide is composed of proline (Pro) and hydroxyproline (Hyp), and its concentration can reflect the synthesis status of collagen [49]. Prolonged UV irradiation can lead to the loss and structural changes in collagen in the skin, resulting in a decrease in Pro-Hyp levels. In this study, following differential metabolite analysis, a significant reduction (*p* < 0.001) in Pro-Hyp was detected in the skin of mice post-UVB irradiation. Conversely, M-Gly intervention resulted in a significant increase (*p* < 0.05) in Pro-Hyp levels within the skin metabolome, further demonstrating that M-Gly intervention can attenuate the degradation of collagen in the skin caused by UVB exposure. KEGG enrichment analysis revealed that M-Gly aids in counteracting UV-induced skin damage by modulating several key metabolic pathways, particularly amino sugar and nucleotide sugar metabolism, linoleic acid metabolism, carbohydrate digestion and absorption, sphingolipid signaling pathway, and choline metabolism in cancer. These pathways collectively support skin immune function, antioxidant capacity, and repair mechanisms, thereby alleviating UV-induced cellular damage, aging, and inflammation, promoting skin health, and potentially reducing the risk of UV-induced skin cancer.

In this study, we also employed transcriptomic technology to examine the differentially expressed genes in mouse skin tissue. M-Gly regulated a total of 752 differential genes. Pathway enrichment analysis of these differential genes revealed that M-Gly may improve skin UV damage through multiple mechanisms, including the regulation of ion transport, enhancement of cellular repair and regeneration capabilities, promotion of metabolic and antioxidant responses, improvement of membrane stability, and modulation of cellular signaling pathways. Through these pathways, M-Gly can effectively alleviate the damage caused by UV radiation and enhance the adaptability and health of skin cells. However, the actual RNA expression changes detected through sequencing are the result of layered negative feedback regulation, and different tissues have varying sensitivities to expression differences. GSEA is a commonly used method in medical research that does not require pre-filtering of genes, thereby avoiding the limitations of traditional enrichment analysis, such as the loss of valuable information from genes with minor effects due to “fixed-threshold” filtering [50,51]. This approach enables a more comprehensive interpretation of the regulatory effects within a specific functional unit. In this study, to elucidate whether the antioxidant M-Gly exerts its pharmacological effects by activating pathways related to oxidative stress, the oxidative stress-related genes within the H_MG_MC gene set were analyzed to identify any significant enrichment in biological pathways or processes that are associated with the effects of M-Gly on UVB-induced skin damage through GSEA method. The results showed a significant difference in the expression of the Nrf2-related pathway between the model group and the M-Gly intervention group, with most gene expressions concentrated in the M-Gly group. qRT-PCR validation of the key genes, Nrf2 and HO-1 mRNA, supported the GSEA result. UV radiation-induced oxidative stress is a critical factor in accelerating skin damage and aging. In this process, the activation of the Nrf2 signaling pathway is essential for regulating the expression of downstream antioxidant enzymes, including HO-1, SOD, and CAT [52]. Under normal physiological conditions, the expression of HO-1 is typically low or virtually non-existent in most cells and tissues [53]. However, in response to robust oxidative stress stimuli, the expression of HO-1 is significantly upregulated in most cells, providing a protective mechanism against oxidative damage [54,55]. Furthermore, the expression of HO-1 is significantly regulated by the Nrf2 signaling pathway [56]. In this study, based on the previously measured oxidative stress indicators, we observed that M-Gly intervention significantly promoted the expression of HO-1 as well as the activity of SOD and CAT, an effect that is likely closely associated with the activation of the Nrf2 signaling pathway.

In previous studies, MAA compounds have been recognized as topical formulations in research on photodamage and photoaging. For example, the topical application of shonorine and porphyra-334, derived from Porphyra yezoensis, has been shown to enhance skin antioxidant enzyme activity and inhibit inflammatory responses, thereby protecting the skin from photodamage [57]. These compounds have also been incorporated into the sunscreen product Helioguard^®^ 365 [58]. In this study, our experiments revealed that the intraperitoneal administration of M-Gly significantly protected mice from UVB-induced skin damage. This suggests that M-Gly could be a crucial endogenous agent, offering protection against oxidative stress-related skin damage. Similarly, studies have demonstrated that intraperitoneal injections of β-Nicotinamide [46], biliverdin [59], Echinochrome A [60], and hydrogen-rich saline [61] can protect against acute skin photodamage. However, further studies are needed to determine the exact molecular mechanisms by which M-Gly acts on different target cells. Additionally, the long-term effects of M-Gly intervention need to be further evaluated, which is crucial for its future clinical application.

## 5. Conclusions

In this study, M-Gly has been demonstrated to exert pharmacological effects within organisms to combat UVB-induced skin photodamage. Our study confirmed that M-Gly possesses robust antioxidant capabilities, which aid in restoring redox balance, preventing collagen degradation, and modulating inflammatory responses in damaged skin. Through metabolomic and transcriptomic analyses, we found that M-Gly intervention regulates key metabolic and genetic pathways critical for skin health. These include pathways for amino sugar, nucleotide sugar, and sphingolipid metabolism, as well as the Nrf2 oxidative stress pathway. These results highlight M-Gly’s potential as a safe, natural antioxidant suitable for development as an active ingredient in UV-protective skincare products, offering a promising strategy to combat skin aging and inflammation induced by UV exposure.

## Figures and Tables

**Table 1:** Animal experimental groups and treatment methods.

Group	Treatment	Period (Days)	UVB Intensity (μW/cm^2^)
NC ^1^	No treatment	30	0
MC ^2^	0.9% Normal saline	30	50
H-MG ^3^	2 mg/mL M-Gly ^5^	30	50
L-MG ^4^	0.2 mg/mL M-Gly	30	50

^1^ Normal Control Group; ^2^ Model Negative Control Group; ^3^ High-Dose Experimental Group; ^4^ Low-Dose Experimental Group; ^5^ Mycosporine-glycine.

**Table 2:** Blood routine test results of mice.

Parameters and Groups	NC	MC	H-MG	L-MG
Leukocyte count (10^9^/L)	4.10 ± 1.65	3.37 ± 0.55	3.23 ± 1.75	2.43 ± 1.15
Lymphocyte count(10^9^/L)	3.17 ± 1.65	2.50 ± 1.14	2.33 ± 1.42	1.57 ± 0.65
Monocyte count (10^9^/L)	0.17 ± 0.12	0.20 ± 0.17	0.13 ± 0.06	0.10 ± 0.10
Neutrophil count (10^9^/L)	0.77 ± 0.12	0.67 ± 0.47	0.77 ± 0.38	0.77 ± 0.40
Lymphocyte percentage (10^9^/L)	74.00 ± 12	72.03 ± 24.32	68.97 ± 14.63	65.17 ± 3.61
Monocyte percentage (10^9^/L)	5.70 ± 4.44	6.60 ± 7.19	4.17 ± 0.81	5.40 ± 1.32
Neutrophil percentage (10^9^/L)	20.30 ± 7.56	21.37 ± 17.14	26.87 ± 14.25	29.43 ± 2.34
Erythrocyte count (10^9^/L)	8.39 ± 0.44	8.56 ± 0.35	8.39 ± 0.12	8.08 ± 0.67
Hemoglobin (10^9^/L)	131.67 ± 7.02	135.67 ± 3.06	130.33 ± 2.52	136.00 ± 13.53
Hematocrit (%)	44.80 ± 2.13	46.73 ± 1.63	44.20 ± 1.25	42.63 ± 3.19
Mean erythrocyte volume (fL)	53.47 ± 0.90	54.67 ± 0.42	52.77 ± 1.54	52.83 ± 1.42
Mean erythrocyte hemoglobin content (pg)	15.63 ± 0.35	15.80 ± 0.26	15.50 ± 0.26	16.77 ± 0.85
Mean erythrocyte hemoglobin concentration (g/L)	293.67 ± 2.31	289.67 ± 3.51	294.33 ± 3.21	318.00 ± 9.54
Erythrocyte distribution width (%)	14.23 ± 1.36	14.07 ± 0.55	15.37 ± 0.15	14.57 ± 0.74
Platelet count (10^9^/L)	1000.33 ± 184.19	1171 ± 175.6	1158 ± 373.48	1072.33 ± 365.43
Mean platelet volume (fL)	6.43 ± 0.51	7.03 ± 0.35	6.97 ± 0.57	6.43 ± 0.12
Platelet distribution width	16.80 ± 0.46	16.80 ± 0.10	16.67 ± 0.12	17.17 ± 0.42

## Data Availability

Dataset available on request from the authors.

## References

[1] Bai Y., Zhao Y., Li Y., Xu J., Fu X., Gao X., Mao X., Li Z. (2020). Uv-Shielding Alginate Films Crosslinked with Fe (3+) Containing Edta. Carbohydr. Polym..

[2] Gu Y., Han J., Jiang C., Zhang Y. (2020). Biomarkers, Oxidative Stress and Autophagy in Skin Aging. Ageing Res. Rev..

[3] Diffey B.L. (1991). Solar Ultraviolet Radiation Effects on Biological Systems. Phys. Med. Biol..

[4] Feng G., Wei L., Che H., Shen Y., Mi K., Bian H., Yang H., Wu J., Mu L. (2022). Cathelicidin-Nv from Nanorana Ventripunctata Effectively Protects Hacat Cells, Ameliorating Ultraviolet B-Induced Skin Photoaging. Peptides.

[5] Lv H., Liu Q., Zhou J., Tan G., Deng X., Ci X. (2017). Daphnetin-Mediated Nrf2 Antioxidant Signaling Pathways Ameliorate Tert-Butyl Hydroperoxide (T-Bhp)-Induced Mitochondrial Dysfunction and Cell Death. Free Radic. Biol. Med..

[6] Lan X., Han X., Li Q., Wang J. (2017). (-)-Epicatechin, a Natural Flavonoid Compound, Protects Astrocytes against Hemoglobin Toxicity Via Nrf2 and Ap-1 Signaling Pathways. Mol. Neurobiol..

[7] Wang J., Fields J., Zhao C., Langer J., Thimmulappa R.K., Kensler T.W., Yamamoto M., Biswal S., Doré S. (2007). Role of Nrf2 in Protection against Intracerebral Hemorrhage Injury in Mice. Free Radic. Biol. Med..

[8] Sun G., Wang J., Xu X., Zhai L., Li Z., Liu J., Zhao D., Jiang R., Sun L. (2023). Panax Ginseng Meyer Cv. Silvatica Phenolic Acids Protect DNA from Oxidative Damage by Activating Nrf2 to Protect Hff-1 cells from Uva-Induced Photoaging. J. Ethnopharmacol..

[9] Wang J., Doré S. (2007). Heme Oxygenase-1 Exacerbates Early Brain Injury after Intracerebral Haemorrhage. Brain.

[10] Zhang Z., Song Y., Zhang Z., Li D., Zhu H., Liang R., Gu Y., Pang Y., Qi J., Wu H. (2017). Distinct Role of Heme Oxygenase-1 in Early- and Late-Stage Intracerebral Hemorrhage in 12-Month-Old Mice. J. Cereb. Blood Flow Metab..

[11] Chen Q.M., Maltagliati A.J. (2018). Nrf2 at the Heart of Oxidative Stress and Cardiac Protection. Physiol. Genom..

[12] Petrova A., Davids L.M., Rautenbach F., Marnewick J.L. (2011). Photoprotection by Honeybush Extracts, Hesperidin and Mangiferin against Uvb-Induced Skin Damage in Skh-1 Mice. J. Photochem. Photobiol. B.

[13] Lautenschlager S., Wulf H.C., Pittelkow M.R. (2007). Photoprotection. Lancet.

[14] Young A.R. (2006). Acute Effects of Uvr on Human Eyes and Skin. Prog. Biophys. Mol. Biol..

[15] Leccia M.T., Lebbe C., Claudel J.P., Narda M., Basset-Seguin N. (2019). New Vision in Photoprotection and Photorepair. Dermatol. Ther..

[16] Yang L.J., Knoll J., Kundu R.V. (2023). Consumer Attitudes toward Aging Skin During the COVID-19 Pandemic. Int. J. Womens Dermatol..

[17] Rosic N.N., Dove S. (2011). Mycosporine-Like Amino Acids from Coral Dinoflagellates. Appl. Environ. Microbiol..

[18] Kageyama H., Waditee-Sirisattha R., Rastogi R.P. (2018). Cyanobacterial Uv Sunscreen: Biosynthesis, Regulation, and Application. Sunscreens: Source, Formulations, Efficacy and Recommendations.

[19] Geraldes V., Pinto E. (2021). Mycosporine-Like Amino Acids (Maas): Biology, Chemistry and Identification Features. Pharmaceuticals.

[20] Wada N., Sakamoto T., Matsugo S. (2015). Mycosporine-Like Amino Acids and Their Derivatives as Natural Antioxidants. Antioxidants.

[21] Ryu J., Park S.J., Kim I.H., Choi Y.H., Nam T.J. (2014). Protective Effect of Porphyra-334 on Uva-Induced Photoaging in Human Skin Fibroblasts. Int. J. Mol. Med..

[22] Suh S.S., Hwang J., Park M., Seo H.H., Kim H.S., Lee J.H., Moh S.H., Lee T.K. (2014). Anti-Inflammation Activities of Mycosporine-Like Amino Acids (Maas) in Response to Uv Radiation Suggest Potential Anti-Skin Aging Activity. Mar. Drugs.

[23] Tarasuntisuk S., Patipong T., Hibino T., Waditee-Sirisattha R., Kageyama H. (2018). Inhibitory Effects of Mycosporine-2-Glycine Isolated from a Halotolerant Cyanobacterium on Protein Glycation and Collagenase Activity. Lett. Appl. Microbiol..

[24] Hartmann A., Gostner J., Fuchs J.E., Chaita E., Aligiannis N., Skaltsounis L., Ganzera M. (2015). Inhibition of Collagenase by Mycosporine-Like Amino Acids from Marine Sources. Planta Medica.

[25] Dunlap W.C., Yamamoto Y. (1995). Small-Molecule Antioxidants in Marine Organisms: Antioxidant Activity of Mycosporine-Glycine. Comp. Biochem. Physiol. B Comp. Biochem..

[26] Suh H.J., Lee H.W., Jung J. (2003). Mycosporine Glycine Protects Biological Systems against Photodynamic Damage by Quenching Singlet Oxygen with a High Efficiency. Photochem. Photobiol..

[27] Idle J.R., Gonzalez F.J. (2007). Metabolomics. Cell Metab..

[28] Horgan R.P., Broadhurst D.I., Walsh S.K., Dunn W.B., Brown M., Roberts C.T., North R.A., McCowan L.M., Kell D.B., Baker P.N. (2011). Metabolic Profiling Uncovers a Phenotypic Signature of Small for Gestational Age in Early Pregnancy. J. Proteome Res..

[29] Elpa D.P., Chiu H.Y., Wu S.P., Urban P.L. (2021). Skin Metabolomics. Trends Endocrinol. Metab..

[30] Wu J., Fang Z., Liu T., Hu W., Wu Y., Li S. (2021). Maximizing the Utility of Transcriptomics Data in Inflammatory Skin Diseases. Front. Immunol..

[31] Balskus E.P., Walsh C.T. (2010). The Genetic and Molecular Basis for Sunscreen Biosynthesis in Cyanobacteria. Science.

[32] Livak K.J., Schmittgen T.D. (2001). Analysis of Relative Gene Expression Data Using Real-Time Quantitative Pcr and the 2(-Delta Delta C(T)) Method. Methods.

[33] Liu H.M., Cheng M.Y., Xun M.H., Zhao Z.W., Zhang Y., Tang W., Cheng J., Ni J., Wang W. (2023). Possible Mechanisms of Oxidative Stress-Induced Skin Cellular Senescence, Inflammation, and Cancer and the Therapeutic Potential of Plant Polyphenols. Int. J. Mol. Sci..

[34] Baek J., Lee M.G. (2016). Oxidative Stress and Antioxidant Strategies in Dermatology. Redox Rep..

[35] Martinez R.M., Pinho-Ribeiro F.A., Steffen V.S., Silva T.C., Caviglione C.V., Bottura C., Fonseca M.J., Vicentini F.T., Vignoli J.A., Baracat M.M. (2016). Topical Formulation Containing Naringenin: Efficacy against Ultraviolet B Irradiation-Induced Skin Inflammation and Oxidative Stress in Mice. PLoS ONE.

[36] Zhou X., Sun H., Tan F., Yi R., Zhou C., Deng Y., Mu J., Zhao X. (2021). Anti-Aging Effect of Lactobacillus Plantarum Hfy09-Fermented Soymilk on D-Galactose-Induced Oxidative Aging in Mice through Modulation of the Nrf2 Signaling Pathway. J. Funct. Foods.

[37] Varnali T., Bozoflu M., Şengönül H., Kurt S.İ. (2022). Potential Metal Chelating Ability of Mycosporine-Like Amino Acids: A Computational Research. Chemical. Pap..

[38] Smith L.T., Holbrook K.A., Madri J.A. (1986). Collagen Types I, Iii, and V in Human Embryonic and Fetal Skin. Am. J. Anat..

[39] Woessner J.F. (1994). The Family of Matrix Metalloproteinases. Ann. N. Y. Acad. Sci..

[40] Orfanoudaki M., Hartmann A., Alilou M., Gelbrich T., Planchenault P., Derbré S., Schinkovitz A., Richomme P., Hensel A., Ganzera M. (2019). Absolute Configuration of Mycosporine-Like Amino Acids, Their Wound Healing Properties and in Vitro Anti-Aging Effects. Mar. Drugs.

[41] Volkmann M., Gorbushina A.A., Kedar L., Oren A. (2006). Structure of Euhalothece-362, a Novel Red-Shifted Mycosporine-Like Amino Acid, from a Halophilic Cyanobacterium (*Euhalothece* sp.). FEMS Microbiol. Lett..

[42] Jain J., Arora S., Rajwade J.M., Omray P., Khandelwal S., Paknikar K.M. (2009). Silver Nanoparticles in Therapeutics: Development of an Antimicrobial Gel Formulation for Topical Use. Mol. Pharm..

[43] Yeh Y.C., Creran B., Rotello V.M. (2012). Gold Nanoparticles: Preparation, Properties, and Applications in Bionanotechnology. Nanoscale.

[44] Singh M., Thakur V., Kumar V., Raj M., Gupta S., Devi N., Upadhyay S.K., Macho M., Banerjee A., Ewe D. (2022). Silver Nanoparticles and Its Mechanistic Insight for Chronic Wound Healing: Review on Recent Progress. Molecules.

[45] Kligman L.H., Murphy G.F. (1996). Ultraviolet B Radiation Increases Hairless Mouse Mast Cells in a Dose-Dependent Manner and Alters Distribution of Uv-Induced Mast Cell Growth Factor. Photochem. Photobiol..

[46] Zhou X., Du H.H., Long X., Pan Y., Hu J., Yu J., Zhao X. (2021). Β-Nicotinamide Mononucleotide (Nmn) Administrated by Intraperitoneal Injection Mediates Protection against Uvb-Induced Skin Damage in Mice. J. Inflamm. Res..

[47] Asai T.T., Miyauchi S., Wijanarti S., Sekino A., Suzuki A., Maruya S., Mannari T., Tsuji A., Toyama K., Nakata R. (2024). Hydroxyprolyl-Glycine in 24 H Urine Shows Higher Correlation with Meat Consumption Than Prolyl-Hydroxyproline, a Major Collagen Peptide in Urine and Blood. Nutrients.

[48] Asai T.T., Oikawa F., Yoshikawa K., Inoue N., Sato K. (2019). Food-Derived Collagen Peptides, Prolyl-Hydroxyproline (Pro-Hyp), and Hydroxyprolyl-Glycine (Hyp-Gly) Enhance Growth of Primary Cultured Mouse Skin Fibroblast Using Fetal Bovine Serum Free from Hydroxyprolyl Peptide. Int. J. Mol. Sci..

[49] Jimi S. (2017). G-Csf Administration Accelerates Cutaneous Wound Healing Accompanied with Increased Pro-Hyp Production in Db/Db Mice. J. Clin. Res. Dermatol..

[50] Abatangelo L., Maglietta R., Distaso A., D’Addabbo A., Creanza T.M., Mukherjee S., Ancona N. (2009). Comparative Study of Gene Set Enrichment Methods. BMC Bioinform..

[51] Aguilar B., Abdilleh K., Acquaah-Mensah G.K. (2023). Multi-Omics Inference of Differential Breast Cancer-Related Transcriptional Regulatory Network Gene Hubs between Young Black and White Patients. Cancer Genet..

[52] Ikehata H., Yamamoto M. (2018). Roles of the Keap1-Nrf2 System in Mammalian Skin Exposed to Uv Radiation. Toxicol. Appl. Pharmacol..

[53] Campbell N.K., Fitzgerald H.K., Dunne A. (2021). Regulation of Inflammation by the Antioxidant Haem Oxygenase 1. Nat. Rev. Immunol..

[54] Gozzelino R., Jeney V., Soares M.P. (2010). Mechanisms of Cell Protection by Heme Oxygenase-1. Annu. Rev. Pharmacol. Toxicol..

[55] Ferrándiz M.L., Devesa I. (2008). Inducers of Heme Oxygenase-1. Curr. Pharm. Des..

[56] Ma Q. (2013). Role of Nrf2 in Oxidative Stress and Toxicity. Annu. Rev. Pharmacol. Toxicol..

[57] Ying R., Zhang Z., Zhu H., Li B., Hou H. (2019). The Protective Effect of Mycosporine-Like Amino Acids (Maas) from Porphyra Yezoensis in a Mouse Model of Uv Irradiation-Induced Photoaging. Mar. Drugs.

[58] Schmidt E.W. (2011). An Enzymatic Route to Sunscreens. ChemBioChem.

[59] Bai B., Liu Y., You Y., Li Y., Ma L. (2015). Intraperitoneally Administered Biliverdin Protects against Uvb-Induced Skin Photo-Damage in Hairless Mice. J. Photochem. Photobiol. B.

[60] Seol J.E., Ahn S.W., Seol B., Yun H.R., Park N., Kim H.K., Vasileva E.A., Mishchenko N.P., Fedoreyev S.A., Stonik V.A. (2021). Echinochrome a Protects against Ultraviolet B-Induced Photoaging by Lowering Collagen Degradation and Inflammatory Cell Infiltration in Hairless Mice. Mar. Drugs.

[61] Guo Z., Zhou B., Li W., Sun X., Luo D. (2012). Hydrogen-Rich Saline Protects against Ultraviolet B Radiation Injury in Rats. J. Biomed. Res..

